# Combining DMI and [^18^F]FDG-PET can complement the assessment of metabolic dysfunction-associated fatty liver disease

**DOI:** 10.1186/s41747-026-00724-z

**Published:** 2026-05-07

**Authors:** Viktoria Ehret, Usevalad Ustsinau, Clemens Fürnsinn, Thomas Scherer, Jana Starčuková, Marcus Hacker, Martin Krššák, Cécile Philippe

**Affiliations:** 1 Department of Medicine III, Division of Endocrinology and Metabolism, Medical University of Vienna, Vienna, Austria; 2 Department of Biomedical Imaging and Image-Guided Therapy, Division of Nuclear Medicine, Medical University of Vienna, Vienna, Austria; 3 Institute of Scientific Instruments, Czech Academy of Sciences, Brno, Czech Republic

**Keywords:** Deuterium, Glucose metabolism disorders, Magnetic resonance spectroscopy, Non-alcoholic fatty liver disease, Positron emission tomography

## Abstract

**Abstract:**

The unique capability of deuterium metabolic imaging (DMI) to detect downstream metabolic products and trace substrates’ transport within tissues using conventional magnetic resonance (MR) scanners can, in theory, be employed with routine positron emission tomography (PET)/MR equipment. Our technical proof-of-concept study proposes a protocol for the simultaneous acquisition of DMI and [¹⁸F]FDG-PET data to enable dual assessment of hepatic glucose metabolism. A protocol that integrates high-dose glucose administration, required for DMI, with [¹⁸F]FDG-PET imaging was applied in a spectroscopy-validated rodent model of metabolic dysfunction-associated fatty liver disease (MAFLD). We acquired and quantified high-quality DMI and PET data of the liver that could provide a distinction between healthy and MAFLD cohorts in the future.

**Relevance statement:**

This proof-of-concept study demonstrates the simultaneous DMI-[^18^F]FDG-PET acquisition for assessing hepatic glucose metabolism. With its proven viability in healthy and MAFLD livers, hybrid DMI-PET imaging shows promise as a prospective non-invasive tool to improve metabolic disease characterization and support future research applications.

**Key Points:**

- Simultaneous DMI and [¹⁸F]FDG-PET acquisition is technically feasible on a standard PET/MR system.
- Hybrid DMI-PET imaging enables dual assessment of hepatic glucose transport and metabolism, offering the advantage of reduced assessment duration.
- High-dose glucose administration for DMI is compatible with PET imaging, thereby reducing the subject’s burden and enhancing liver metabolic evaluation.

**Graphical Abstract:**

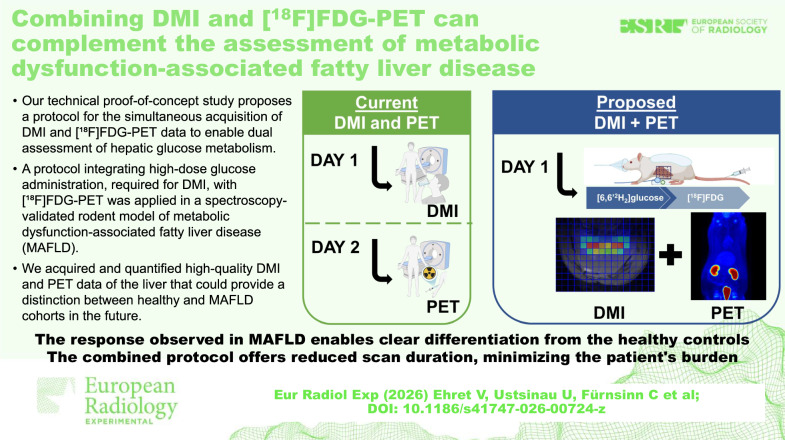

## Background

Positron emission tomography (PET) assesses metabolic pathways and is essential for investigating disease-related changes in energy metabolism. However, PET detects only the radiolabel, not the chemical species, limiting assessment of downstream metabolism.

Deuterium metabolic imaging (DMI), a magnetic resonance spectroscopy (MRS)-based technique, addresses this by providing complementary metabolic information [1, 2]. By administering deuterated substrates and integrating with conventional ¹H magnetic resonance (MR) imaging for anatomical localization, DMI enables spatial mapping and absolute rather than relative quantification of *in vivo* metabolites such as glutamine/glutamate, lipids, acetate, glucose, or deuterated water, along with the downstream products. It is robust to magnetic field inhomogeneities and relatively easily implementable in clinical settings with appropriate hardware [3, 4].

Previously [5], the turnover of [6,6’-²H₂]glucose and [^18^F]fluorodeoxyglucose ([^18^F]FDG) has already been studied in pigs during rapid co-injection through the femoral vein, showing equal plasma disappearance rates after fasting. Additionally, recent assumptions [6] propose that the presence of glucose can be the primary stimulus for hepatic glucose uptake during [^18^F]FDG-PET measurement, and consequently, glucose load should maximize it and improve the quality of liver imaging. In our previous study [7], we examined hepatic glucose and palmitic metabolism in rats with and without metabolic dysfunction-associated fatty liver disease (MAFLD) using DMI alone. Current technical settings allow combined DMI-PET protocols to assess hepatic metabolism and MAFLD-related alterations. The key objective of the present study was to assess the simultaneous acquisition and the influence of a high load of deuterated glucose on hepatic [^18^F]FDG-PET imaging and quantification.

## Methods

### Animals

A metabolic prospective study was conducted in lean and obese male Sprague Dawley rats (Table S1). Four-week-old male rats (Janvier Laboratories) were randomly assigned to two dietary groups. The standard diet (SD) group (*n* = 6) received a carbohydrate-rich chow (LASQCdiet Rod16, Altromin), while the high-fat diet (HFD) group (*n* = 6) was fed a diet providing 60% of calories from fat (Research Diets D12492, New Brunswick, NJ, USA). Rats were housed at 22°C under a 12:12-h light-dark cycle.

All animal procedures adhered to the European Commission Directive 2010/63/EU, ARRIVE [8], and FELASA [9] guidelines for animal research. They were approved by the Austrian Federal Ministry of Science, Research, and Economy (license number: BMBWF 2020-0.078.441).

### Phenotyping

After 6 weeks on a diet, an intraperitoneal glucose tolerance test was performed. Following an overnight fast, rats for DMI-PET received an intraperitoneal injection of a 33% (wt/vol) glucose solution (1 g/kg body weight). Blood glucose was measured from the tail tip using a handheld glucometer (GlucoMen Areo, EMRA-MED Arzneimittel GmbH) at four time points: pre-injection, followed by 30 min intervals post-injection.

Liver fat content was quantified using a single-voxel MRS protocol with short echo time stimulated echo acquisition mode (echo time = 5.5 ms, repetition time = 3000 ms, flip angle = 69.5°, number of averages = 64, spectral points = 2048, voxel of interest = 6 × 6 × 6 mm³, bandwidth = 7.9 kHz, respiratory-gated, acquisition time = 3.2 min), as previously described [7].

### DMI-[^18^F]FDG-PET measurements

Metabolic imaging was conducted after 6 weeks on the respective diet in a subset of three animals per group (SD, HFD). Animals were fasted overnight (12‒16 h), anesthetized with inhalative isoflurane (2‒3%), and maintained at ~37 °C using a heating pad. Vital parameters were continuously monitored (SA Instruments) using a breathing sensor (Graseby®) positioned beneath the abdomen. Blood glucose was measured from the tail tip immediately before and after anesthesia.

DMI-[^18^F]FDG-PET was conducted on a 9.4 T Biospec 94/30 MR system with a µPET insert operating on Paravision 360.3.3 (Bruker Biospin). A dual-tuned ^2^H/^1^H surface radiofrequency coil (Ø = 40 mm, Rapid) was positioned over the abdominal region. Animals were placed in a prone position with the liver aligned with the coil’s sensitive region. To optimize magnetic field homogeneity, B_0_ shimming was performed before DMI acquisition.

#### DMI protocol

[6,6’-²H₂]glucose (Sigma-Aldrich) was dissolved in 1 mL 0.9% NaCl (B. Braun) to a final concentration of 1.78 M. A 0.65 g/kg body weight bolus was administered intraperitoneally to overnight-fasted animals immediately before DMI. Anatomical reference ^1^H MR images were obtained using an axial T_1_-weighted fast low-angle shot sequence (repetition time = 30 ms, flip angle = 70°, number of averages = 20, matrix = 120 × 120) under respiratory gating, maintaining an identical field of view as for DMI. Baseline scans were performed before substrate administration, including both non-localized and localized acquisitions. Non-localized spectra, acquired with a pulse-acquire sequence (radiofrequency block pulse with a flip angle of 61.6°, 0.112 ms, repetition time = 400 ms, number of averages = 84, 2048 points, 5.21 kHz bandwidth, 2.5 min acquisition), served as rapid experimental controls. Localized data were obtained using a three-dimensional chemical shift imaging sequence (repetition time = 100 ms, flip angle = 61.6°, number of averages = 36, 512 points, matrix = 12 × 12 × 8 mm³, field of view = 50 × 36 × 20 mm³, 6.06 kHz bandwidth, 10.3 min acquisition). To reduce motion artifacts, 10-mm transverse saturation slabs were applied over the heart and abdominal organs caudal to the liver. A Hamming-weighted k-space scheme was used to improve signal-to-noise ratio. Post-injection, non-localized and localized DMI measurements were acquired in eight consecutive blocks without respiratory gating.

#### [^18^F]FDG-PET protocol

**Fig. 1:**
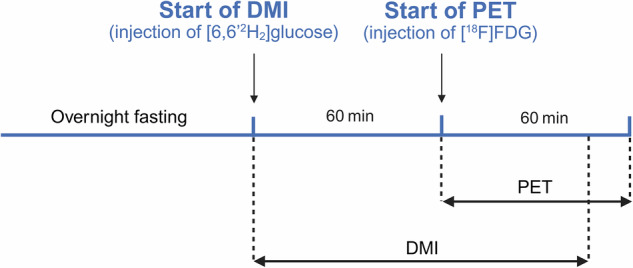
Experimental timeline during combined DMI-[^18^F]FDG-PET measurement

60 min after intraperitoneal [6,6’-^2^H_2_]glucose injection, [^18^F]FDG (24.19 ± 2.37 MBq) was administered intravenously via a tail vein catheter, followed by a 60-min dynamic PET scan (Fig. 1) with frames of 10 s × 6, 20 s × 1, 30 s × 7, 45 s × 1, 60 s × 4, 330 s × 1, and 600 s × 4.

### Control [^18^F]FDG-PET measurements

Overnight-fasted animals (SD = 6, HFD = 6) were positioned on the same PET/MR scanner with a rat PET coil (Ø = 86 mm, Bruker Biospin). [^18^F]FDG (27.95 ± 1.12 MBq) was administered intravenously, followed by a 60 min dynamic PET scan.

To investigate the impact of a high glucose load on [^18^F]FDG-PET, overnight-fasted SD rats received either 1 g/kg body weight D(+)-glucose monohydrate (Sigma-Aldrich) or the equivalent volume ( ~ 2 mL) of physiological saline solution (*n* = 3/group) intravenously. Thirty minutes after the injections, all rats were injected intravenously with [^18^F]FDG (27.13 ± 4.21 MBq) and underwent 60 min PET/MR scanning. Blood glucose was measured at each step.

### Image analysis

**Fig. 2:**
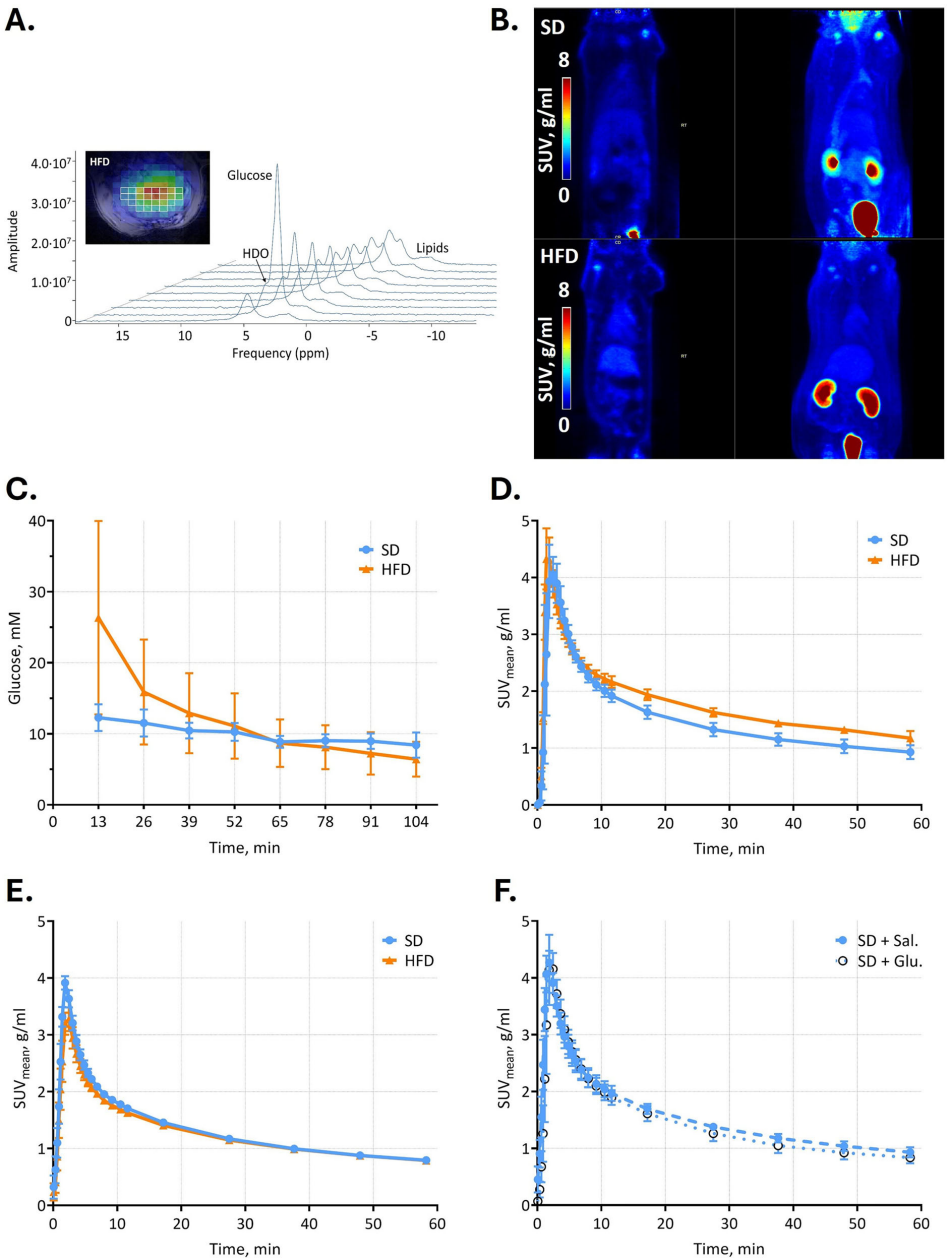
**A** Representative DMI spectra; **B** representative coronal PET images and their maximum intensity projections; **C** hepatic absolute glucose concentration from DMI signal; **D** hepatic SUV_mean_ curves during DMI-[^18^F]FDG-PET; **E** control hepatic SUV_mean_; **F** control hepatic SUV_mean_ after injections of saline (Sal) or glucose (Glu) solution

MRS data were pre-processed and analyzed using jMRUI (v7.0) [10]. Hepatic voxels were manually selected from the chemical shift imaging grid based on radiofrequency coil alignment, sufficient signal-to-noise ratio, and at least one voxel distance from subcutaneous fat to minimize contamination (Fig. 2A). Selected spectra were frequency-aligned and averaged to yield a representative spectrum per time point.

Averaged spectra were quantified by linear least-squares fitting, with amplitudes converted to absolute concentrations (mM) using the natural abundance of the ²H water peak and corrected for baseline hepatic glucose or lipid signals and fat interference, as previously described [7].

PET scans were reconstructed using the MLEM algorithm with 20 iterations (Fig. 2B). The region of interest was drawn along the whole liver in PMOD 4.404 (PMOD Technologies LLC) without preprocessing. Time-activity curves were expressed as standardized uptake values (SUV_mean_).

### Statistical analysis

Animal characteristics and intrahepatic metabolite concentrations are reported as mean ± standard error of the mean. Statistics were calculated via an unpaired *t*-test, and the effect size was determined. For time-course experiments, the area under the curve (AUC) was calculated using the trapezoidal rule to quantify the overall response over time; higher AUC indicates greater overall metabolite concentration (or physiological response) during the observation period.

## Results

### Phenotyping

The phenotypes of healthy and MAFLD animals were confirmed by ^1^H MRS (*p* = 0.010, Table 1). Glucose tolerance test (AUC_total_ *p* = 0.117) and blood glucose are presented in Table 1.

**Table 1:** Phenotyping parameters of the animals

DMI-[^18^F]FDG-PET				
	Standard diet	High-fat diet	*p*-value	Effect size
Mass, g	450.9 ± 23.6	490.8 ± 44.5	NS	1.69
Intrahepatic fat content, %	1.6 ± 0.9	8.6 ± 1.9	0.010	7.78
GTT_,_ mg/dL	131.7 ± 1.2	146.0 ± 3.2	0.014	
60 min	129.7 ± 1.2	137.7 ± 4.3	0.150	6.89
90 min				3.84
Baseline blood glucose, mg/dL	106.3 ± 5.3	114.0 ± 8.8	NS	1.44
Blood glucose after DMI-PET, mg/dL	112.7 ± 53.5	251.7 ± 38.4	0.041	2.60

Control [^18^F]FDG-PET				
	Standard diet	High-fat diet	*p*-value	Effect size
Baseline blood glucose, mg/dL	102.7 ± 3.7	142.5 ± 19.6	NS	4.44
Blood glucose after PET, mg/dL	111.2 ± 4.0	181.3 ± 28.5	0.034	7.21

Control [^18^F]FDG-PET with saline or glucose injection				
	Standard diet + saline	Standard diet + glucose	*p*-value	Effect size
Baseline blood glucose, mg/dL	88.0 ± 1.5	110.7 ± 1.8	< 0.001	8.57
Blood glucose after saline/glucose injection (-30 min), mg/dL	96.3 ± 10.7	499.0 ± 66.9	< 0.001	21.96
Blood glucose at start of PET (0 min), mg/dL	115.3 ± 10.4	207.3 ± 44.5	NS	5.24
Blood glucose after PET (60 min), mg/dL	124.0 ± 12.9	183.0 ± 32.3	NS	2.64

*NS* Not significant, *GTT* Glucose tolerance test

### DMI-[^18^F]FDG-PET

#### DMI quantification

Following [6,6’-^2^H_2_]glucose injection, glucose uptake was similar between groups (AUC_SD_ 902.7 ± 68.9, AUC_HFD_ 1044.0 ± 297.6, *p* = 0.764) (Fig. 2C), and no glycogen signal was detected. The DMI-typical increase of deuterated water over time was noticed in the HFD group with an increase of 46.8 ± 0.6%.

#### [^18^F]FDG-PET quantification

Liver SUV_mean_ curves during DMI-[^18^F]FDG-PET differed between SD and HFD groups (Fig. 2D). Averaged uptake over the last 40 min (4 × 600 s) showed higher SUV_mean_ in HFD rats (1.50 ± 0.04 g/mL) compared with SD (1.11 ± 0.12 g/mL, *p* = 0.034).

### Control [^18^F]FDG-PET

Liver SUV_mean_ time-activity curves did not differ between SD and HFD groups in regular [^18^F]FDG-PET (SUV_mean_, 40 min: SD 0.96 ± 0.02 g/mL *versus* HFD 0.95 ± 0.02 g/mL; Fig. 2E). Further, in SD rats, comparison of glucose *versus* saline injections (Fig. 2F) showed slightly lower uptake (not significant) with glucose (SUV_mean_, 40 min: glucose load 1.02 ± 0.12 g/mL *versus* saline 1.13 ± 0.08 g/mL, *p* = 0.473).

## Discussion

We established a method for simultaneous DMI-[^18^F]FDG-PET measurements. In this hybrid imaging protocol, the DMI signal was recorded after intraperitoneal injection of [6,6’-^2^H_2_]glucose, followed 60 min later by an intravenous administration of [¹⁸F]FDG and the start of PET imaging, enabling concurrent acquisition of both signals.

**Fig. 3:**
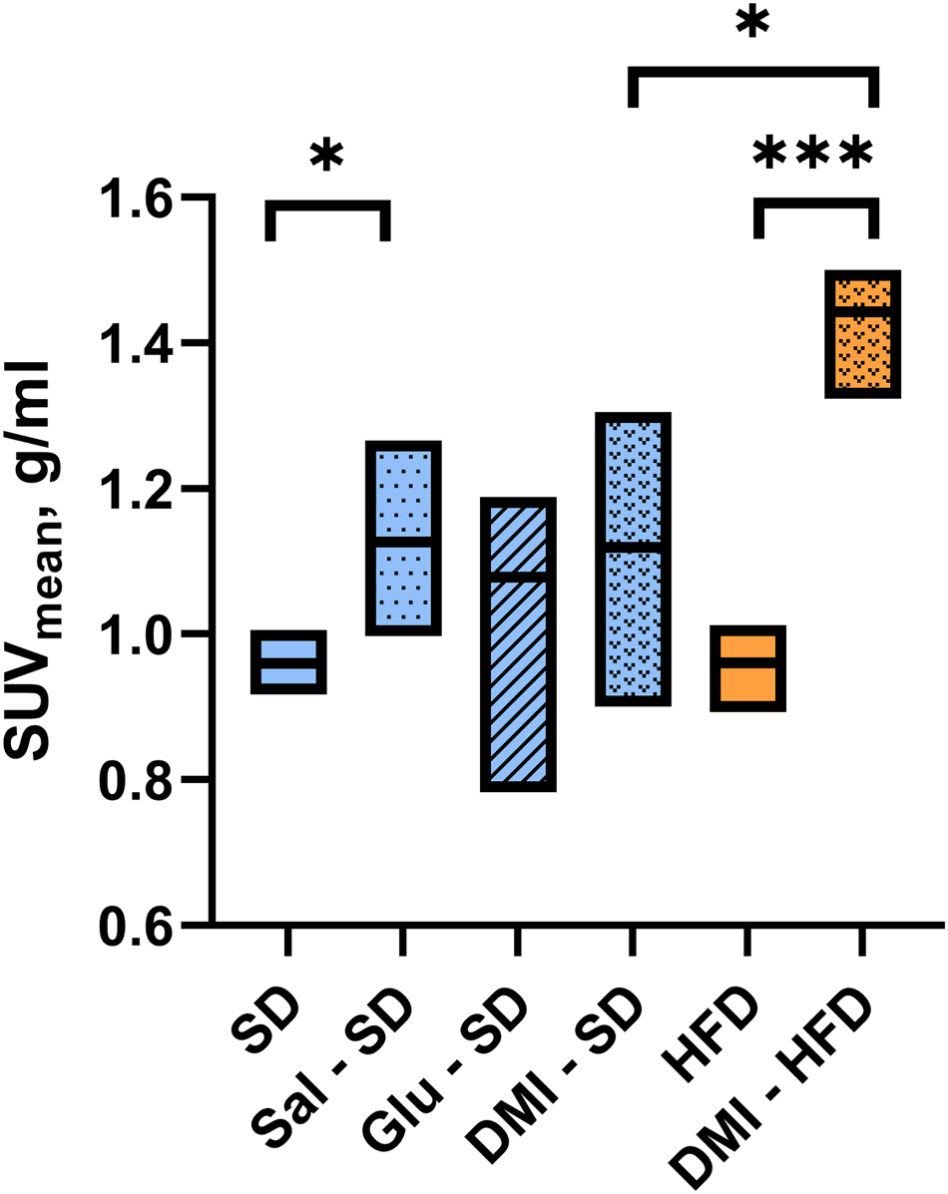
Overall comparison of [^18^F]FDG uptake: standard diet (SD) group, blue; high-fat diet (HFD), orange. *DMI* Injected deuterated glucose, *Glu* Injected glucose, *Sal* Injected saline

In our combined measurement, [¹⁸F]FDG-PET revealed increased hepatic tracer uptake in MAFLD rats. This effect was absent in control PET measurements performed without a glucose load. Additional experiments conducted with saline and glucose load on the healthy cohort demonstrated a modest increase in glucose uptake (Fig. 3). However, it remains difficult to discern the extent to which this uptake is attributable to the injection procedure itself (in case of saline solution) *versus* the physiological response to the injected solution (glucose). The evaluation of more metabolically sensitive MAFLD livers with various injections was beyond the scope of this technical investigation and, therefore, not conducted.

Interpreting the DMI results, it is worth mentioning that glycogen emerges as a downstream product of glucose metabolism, and its detection in glucose DMI is inherently challenging due to rapid isotopic dilution, relatively low glycogen turnover on the time scale of the acquisition, and limited sensitivity of the glycogen resonance under the presented experimental conditions [11, 12]. The absence of detectable downstream signals in this study should therefore not be interpreted as a lack of metabolite synthesis, but as a limitation of our current implementation. Future studies with an appropriate experimental setup may identify metabolite-specific alterations detectable by DMI. In this respect, further combination with ^13^C glucose labeling and concomitant measurements of glycogen accumulation could add further variables for the metabolic framework. Technical potential for such an experimental set-up has already been demonstrated in pivotal multinuclear dynamic MRS studies of human hepatic metabolism applying triple tuned ^1^H/^2^H/^13^C Tx/Rx radiofrequency coil, stable isotope dilution analysis, and metabolic modeling [13, 14].

Consistent with our hypothesis, the most pronounced effect was observed in the MAFLD cohort, with a substantial ~50% increase in uptake (Fig. 3). Current research uses both [6,6’-^2^H_2_]glucose and [^18^F]FDG-PET to assess hepatic glucose uptake in steatosis [15], but on separate measurements. Previous studies have also demonstrated the clinical applicability of DMI [4, 15]. Despite the limited spatial resolution and signal-to-noise ratio, higher field strengths and advanced pulse sequences can improve both within clinically acceptable scan times [16, 17]. Combining DMI and PET in a single procedure could reduce scan time and patient burden while considerably expanding the obtained information. In fact, ultra-high-field scanners (> 7 T) are not yet widely available in routine clinical practice; translation to clinical DMI-PET remains questionable in the near future. Technically, integrating DMI with PET remains promising: while PET alone cannot distinguish metabolites from the parent tracer, the complementary use of DMI enables the detection of downstream metabolites, thereby improving the accuracy of metabolic analysis.

This proof-of-concept study proposes the simultaneous imaging of DMI and [^18^F]FDG-PET. Relying on a high glucose load due to DMI requirements, we were able to establish a combined method for the metabolic evaluation and collect both DMI and [^18^F]FDG-PET data, potentially supporting the research and investigation of MAFLD and related metabolic disorders.

## Supplementary information

**Additional file 1**: **Table S1** Protocols and number of animals employed in the study.

## Data Availability

Data are available upon request.

## Abbreviations

AUC: Area under the curve
DMI: Deuterium metabolic imaging
FDG: Fluorodeoxyglucose
HFD: High-fat diet
MAFLD: Metabolic dysfunction-associated fatty liver disease
MR: Magnetic resonance
MRS: Magnetic resonance spectroscopy
PET: Positron emission tomography
SD: Standard diet
SUV: Standardized uptake value
