# Supplementary material for: Combining DMI and [18F]FDG-PET can complement the assessment of metabolic dysfunction-associated fatty liver disease
Source: Eur Radiol Exp. 2026 May 7;10:62. doi: 10.1186/s41747-026-00724-z (PMC13153327; doi:10.1186/s41747-026-00724-z)
Supplement: Supplementary file 1 — Additional file 1: Table S1 Protocols and number of animals employed in the study. [file 41747_2026_724_MOESM1_ESM.pdf]

Combining DMI and [18F]FDG-PET can complement the assessment of metabolic dysfunction-associated fatty liver disease

ELECTRONIC SUPPLEMENTARY MATERIAL

Table S1. Protocols and number of animals employed in the study

Protocol			$[^{18}F]$FDG-PET scans	
	**Route**	**Dose**	**SD**	**HFD**
DMI	i.p.	0.65 g/kg	3	3
No additional injection (control)	-	-	6	6
Saline (Sal)	i.v.	~3 ml/kg	3	Not performed
Glucose (Glu)	i.v.	1 g/kg	3	Not performed
